# De novo progressive dural arteriovenous fistula with putamen hemorrhage associated with long-term Down syndrome: A case report and literature review

**DOI:** 10.1016/j.radcr.2023.04.009

**Published:** 2023-04-29

**Authors:** Fukutaro Ohgaki, Jun Suenaga, Nobuyuki Shimizu, Tetsuya Yamamoto

**Affiliations:** Department of Neurosurgery, Yokohama City University Graduate School of Medicine, 3-9 Fukuura, Kanazawa-ku, Yokohama, Kanagawa 236-0004, Japan

**Keywords:** Dural arteriovenous fistula, Venous thrombosis, Chronic hypoxemia, Down syndrome, Eisenmenger syndrome

## Abstract

Dural arteriovenous fistula (DAVF) is considered an acquired change in blood flow related to factors such as craniotomy, trauma, and infection. However, several factors related to its development remain unknown. Here, we present a case of a 48-year-old man with Down syndrome and Eisenmenger syndrome. He had a history of craniotomy for multiple brain abscesses, followed by the occurrence of a de novo straight sinus (StS) DAVF within the last 2 years. The patient presented with right putamen hemorrhage due to venous congestion by a StS DAVF. The shunt flow was occluded by transarterial embolization using Onyx. Several studies have reported on DAVF models induced by venous congestion and hypoxemia. In this case, local venous congestion due to craniotomy for multiple brain abscesses was considered as one of the causes of DAVF. Complication of venous thrombosis or chronic hypoxemia due to Eisenmenger syndrome might have led to its progression. Especially in DAVF cases with Down syndrome, concomitant symptoms such as hypoxemia due to congenital heart failure and coagulopathy could worsen the disease state progressively.

## Introduction

Dural arteriovenous fistula (DAVF) results from an acquired blood flow change and is associated with factors such as craniotomy, trauma, infection, and metabolic factors. However, its etiology remains unknown [1], [2], [3], [4], [5], [6], [7], [8], [9], [10], [11], [12], [13]. The opening of physiological dural arteriovenous channels because of increased venous pressure with obstruction, such as thrombosis [1,^4^,^13^,14], and hypoxemia due to local perfusion degradation with increased venous pressure inducing angiogenesis are considered to be related [4,^6^,^7^,[10], [11], [12], as demonstrated in animal experiments [1,^4^,^6^,^7^,^11^,12]. Here, we present a case of de novo progressive straight sinus (StS) DAVF that occurred within 2 years of craniotomy for brain abscess treatment, and presented with a right putamen hemorrhage in a patient with Down syndrome and Eisenmenger syndrome. We also review relevant literature.

## Case report

A 48-year-old man with a history of Down syndrome, ventricular septal defect (VSD), and Eisenmenger syndrome underwent craniotomy for multiple brain abscesses 2 years earlier. He resided in a nursing home, but despite his intellectual disability due to Down syndrome, he could independently perform most daily living activities. He presented with altered consciousness and left hemiparesis upon waking and, was transferred to our hospital.

**Fig. 1:**
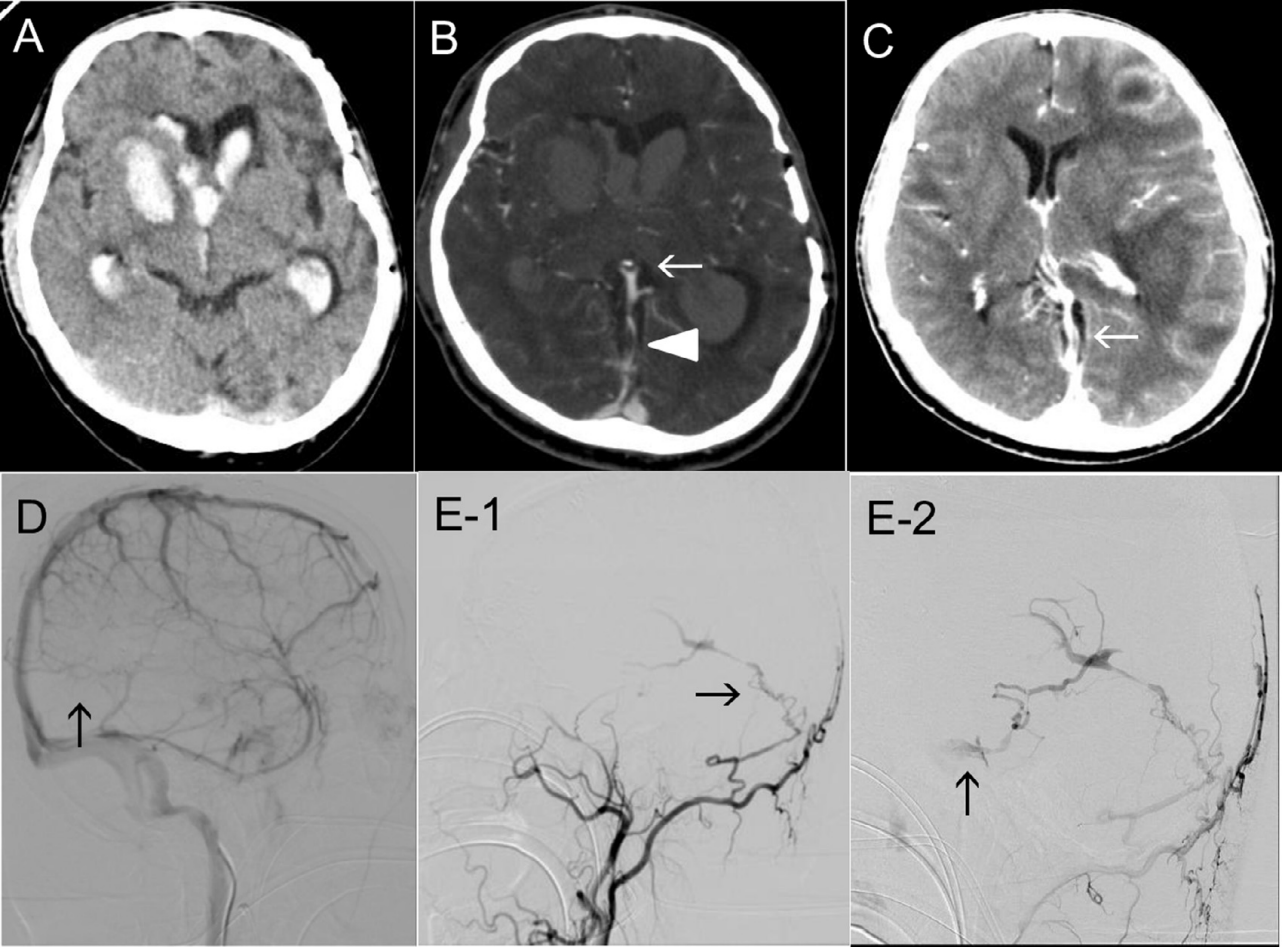
(A) Axial CT on admission showing a right putaminal hemorrhage and acute hydrocephalus due to an intraventricular hematoma. (B) Axial CT angiography image showing the bilateral basal vein and the straight sinus (StS) in the arterial phase (arrow), indicating an arteriovenous shunt. Additionally, contrast CT showing a partial defect of the StS (arrowhead), indicating sinus thrombosis. (C) Contrast-enhanced axial CT at the time of multiple brain abscesses 2 years earlier showing patency of the StS (arrow). (D) Venous phase of right ICAG showing StS occlusion (arrow). (E) Left ECAG showing DAVF shunt point from the mastoid branch of the OA to the StS (E-1, arrow). The venous reflux flows to the left cavernous sinus through the basal vein and uncal vein (E-2, arrow) (E-1, E-2: lateral view). CT, computed tomography; DAVF, dural arteriovenous fistula; ECAG, external carotid artery angiography; ICAG, internal carotid artery angiography; OA, occipital artery; StS, straight sinus.

On admission, the patient presented with a Glasgow Coma Scale (GCS) score of 9(E3V1M5), left hemiparesis (manual muscle testing, MMT 2), and right conjugate eye deviation. Laboratory test results indicated chronic hypoxemia (Hb 22.7 g/dL, pO_2_ 59.7 mmHg, sO_2_ 86.6%), with no evidence of coagulopathy (PT-INR 1.22, APTT 29.6 s, Fib 269 mg/dL, D-dimer 0.79 µg/mL). Electrocardiography and echocardiography showed right heart failure due to Eisenmenger syndrome (incomplete right bundle branch block, ejection fraction 40%-45% [sight], mild tricuspid regurgitation [max pressure gap 83 mmHg], VSD, and bilateral shunt flow). Computed tomography (CT) revealed right putamen hemorrhage, intraventricular hematoma, and acute obstructive hydrocephalus (Fig. 1A). Additionally, an arteriovenous shunt and venous thrombosis were suggested on CT angiography and venography (CTA/V) that were performed to locate the sources of the cerebral hemorrhage (Fig. 1B). This patient had a history of multiple brain abscesses that were treated with craniotomy and antibiotics 2 years earlier. At that time, there was no evidence of sinus thrombosis on contrast-enhanced CT (Fig. 1C).

We initially removed the intraventricular hematoma from the right anterior horn using a rigid endoscope and indwelled ventricular drainage tube under the soft endoscopic guidance. Hydrocephalus caused by the intraventricular hematoma occlusion was controlled, and the drainage tube was removed one week postoperatively. We performed cerebral angiography to locate the source of the hemorrhage. We diagnosed an intracerebral hemorrhage due to venous congestion by StS thrombosis (Fig. 1D) caused by StS DAVF (Cognard type III, Borden type III) with deep vein reflux (Fig. 1E).

**Fig. 2:**
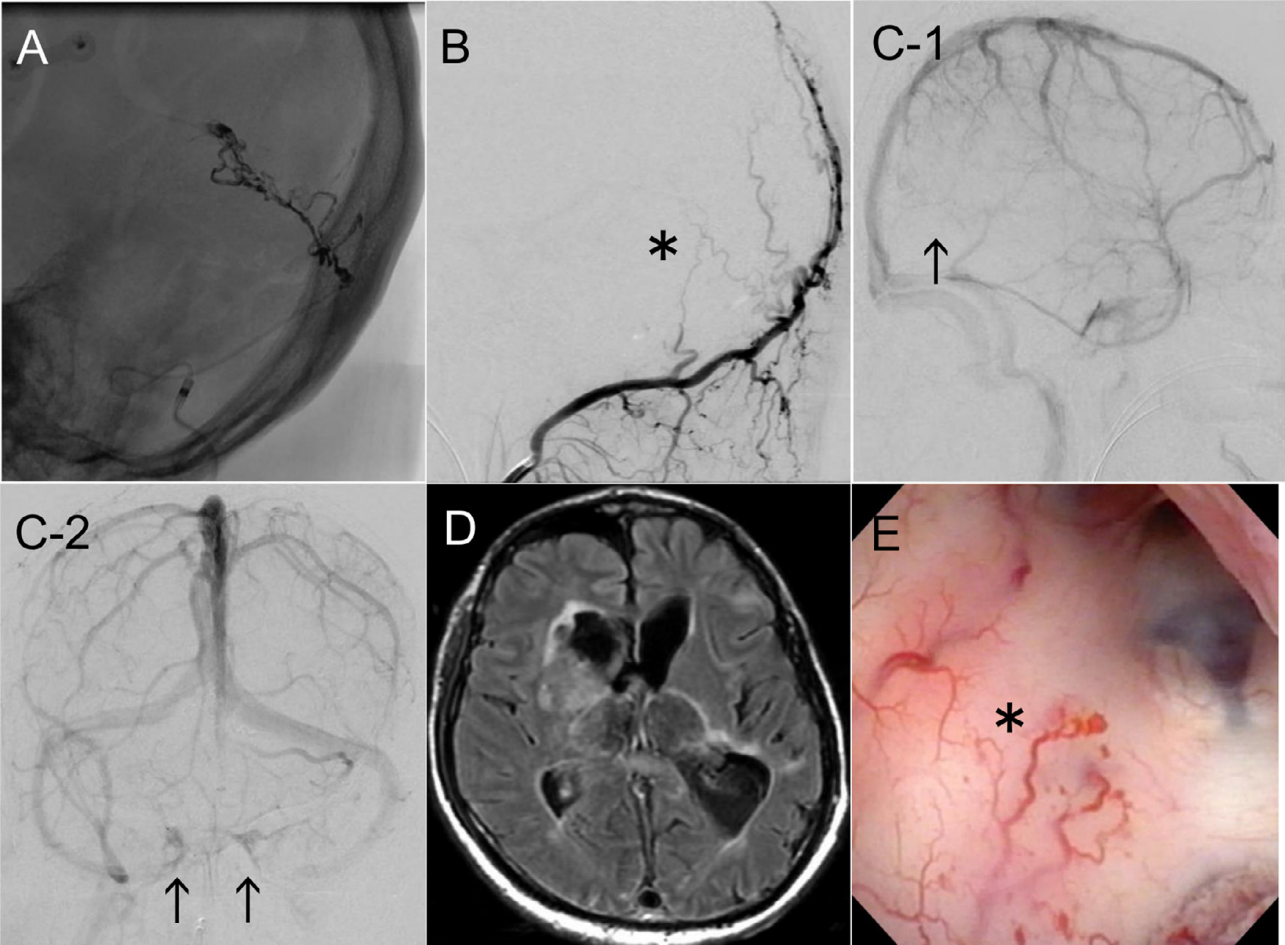
(A) TAE was performed for the shunt point occlusion. Injection from Scepter XC cannulated to the left OA shows shunt flow from the mastoid branch to the StS. Onyx 18 was injected intermittently with balloon inflation, a total of 1.6 mL in 10 minutes. (B) Arteriovenous shunt occlusion was confirmed on postembolization left OA angiography (asterisk, lateral view). (C) Postembolization right ICAG showing remnant of StS occlusion (C-1, arrow), and patency of CS as venous drainage route (C-2, arrow). (D) MRI FLAIR showing bilateral thalamus injury due to high venous pressure by StS DAVF. (E) Intraoperative neuroendoscopy showing hypervascularity on the ventricular wall (asterisk), suggesting congestion of venous return or the effect of angiogenesis. CS, cavernous sinus; DAVF, dural arteriovenous fistula; ICAG, internal carotid artery angiography; MRI FLAIR, magnetic resonance fluid-attenuated inversion recovery imaging; OA, occipital artery; StS, straight sinus; TAE, trans-arterial embolization.

We performed trans-arterial embolization (TAE) of the StS DAVF. We cannulated the left occipital artery (OA) using the 7 Fr FUBUKI 100 cm (Asahi Intec, Aichi, Japan) / 4.2 Fr FUBUKI 125 cm guiding catheter (Asahi Intec, Aichi, Japan), and identified the feeding artery from the mastoid branch of the OA using the Scepter XC 4 × 11 mm balloon catheter (Terumo, Tokyo, Japan) / CHIKAI 14 200 cm micro guide wire (Asahi Intec, Aichi, Japan). We gradually injected 1.6 mL of the Onyx 18 liquid embolic agent (ev3, Tokyo, Japan) over 10 minutes with repeated pauses under the controlled flow using Scepter XC balloon catheter (Fig. 2A). We confirmed that the shunt to the StS was completely occluded through the left occipital artery angiography (OAG) (Fig. 2B). DAVF shunt flow occlusion using TAE was successful, but StS remained to be occluded (Fig. 2C-1). Although other venous drainage routes to the cavernous sinus were suggested to be patent (Fig. 2C-2), altered consciousness persisted due to irreversible bilateral thalamus damage caused by venous congestion from the early period of the clinical course with chronic hematoma (Fig. 2D). Considering the patient's persistent altered consciousness and modified Rankin scale (mRS) score of 5, he was transferred to another hospital.

## Discussion

### Development of DAVF in the present case

**Fig. 3:**
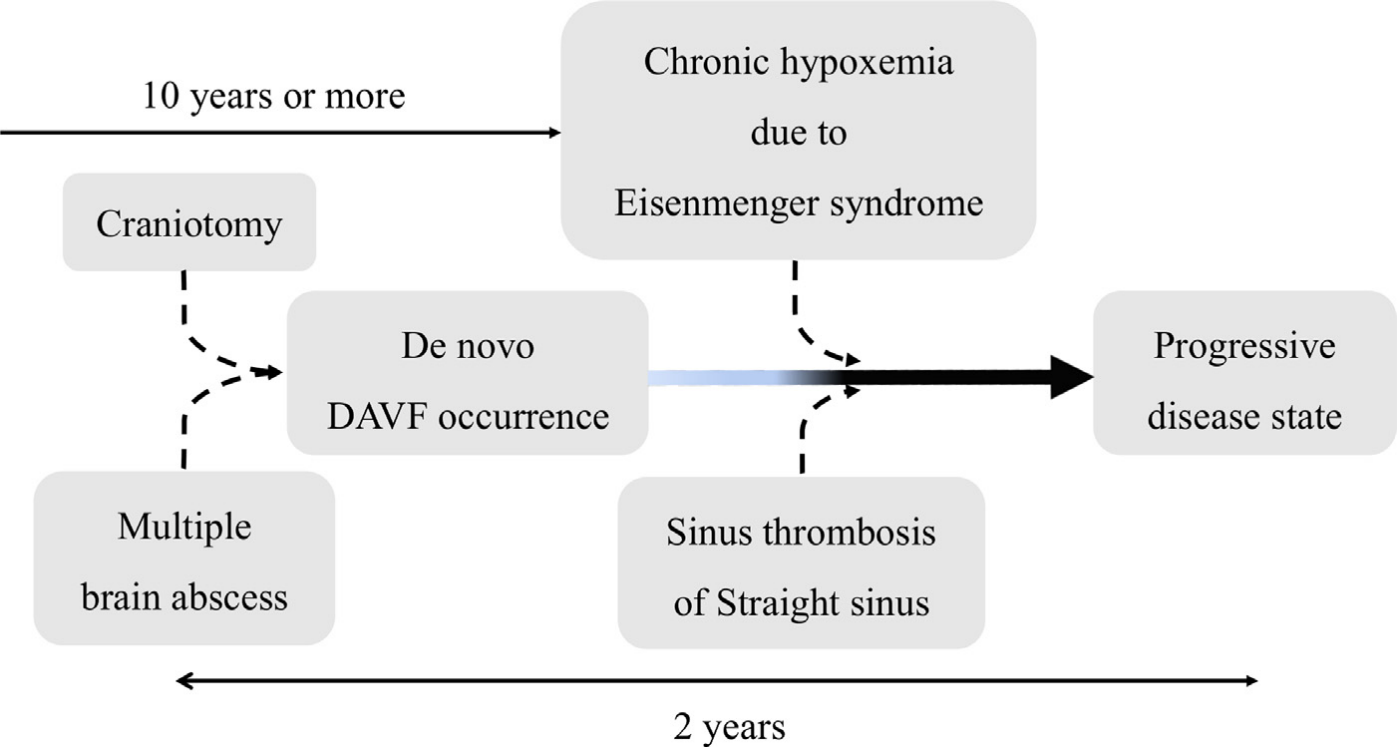
This patient's DAVF might have appeared as a result of infection and craniotomy. Additionally, the DAVF might have progressed due to venous congestion due to venous thrombosis of the straight sinus and the patient's chronic hypoxemic condition with Down syndrome and Eisenmenger syndrome. DAVF, dural arteriovenous fistula.

Three cases of DAVF complicated with Down syndrome have been reported previously [3,14,15], making this the fourth reported case. DAVF is thought to develop due to an acquired blood flow change associated with craniotomy [3,5,8], trauma [8], and infection [2]. Local venous thrombosis and an increase in venous reflux pressure occur concurrently. The compensatory expansion of the original dural arteriovenous channel is related to the occurrence of DAVF [1,4,7]. However, expanding the channel alone is considered insufficient for DAVF occurrence [1,4,6,7,10,11]. Venous congestion leads to local hypoperfusion and increased internal vascular pressure, hypoxemia, and tissue ischemia. As a result, the expressions of vascular endothelial growth factor, basic fibroblast growth factor, and hypoxia-inducible factor 1-alpha are induced, thus leading to angiogenesis which might cause a de novo DAVF [1,3,[6], [7], [8],[10], [11], [12]]. If DAVF occurs, venous reflux pressure increases, and accelerates the progression of the disease state through activation of angiogenesis [2,4,6,9,11,12]. In our case, vascular proliferation in the ventricular wall was remarkable (Fig. 2E) and was considered to be the effect of venous congestion or angiogenesis. No contrast defect in the StS on contrast CT was detected at the time of the brain abscess (Fig. 1C). Therefore, the occurrence of a recent thrombosis or stenosis was suspected, at least within the last 2 years. Given his history of craniotomy for brain abscess treatment, the patient may have developed DAVF de novo as a result of infection and craniotomy. Additionally, DAVF might have progressively worsened due to his Down syndrome and Eisenmenger syndrome with the associated hypoxemic condition (Fig. 3).

### Association of the Down syndrome feature with DAVF development

Down syndrome can be complicated by congenital heart disease, and cerebral vascular disease, and coagulation abnormality. Koduri et al. [3] presented an asymptomatic bilateral DAVF case that was appeared four months after indirect blood flow reconstruction of a left middle cerebral artery occlusion in a patient with Moyamoya disease and Down syndrome. Although it was not complicated with venous thrombosis, further angiogenesis that often occurs in Moyamoya disease could have been promoted by the indirect blood flow reconstruction, in addition to the craniotomy.

Chronic hypoxemia may be associated with the progression of Eisenmenger syndrome, which may subsequently lead to further angiogenesis as in this case. Additionally, venous thrombosis due to a coagulation abnormality may lead to further deterioration and progressive worsening of the disease state. Kim et al. [15] presented a case that was positive for anti-cardiolipin antibody. Berryman et al. [14] presented cases with a Leiden abnormality of coagulant factor V and protein S deficiency, as well as venous thrombosis caused by coagulation abnormality, progressive headaches, visual impairment, and convulsions and venous thrombosis caused by coagulation abnormality. These symptoms appeared in the last several months to a year in those cases. Further progression of venous thrombosis and symptoms caused by venous congestion due to DAVF were also observed. Transvenous embolization (TVE) or TAE was performed in each DAVF case, and the symptoms were improved (Table 1). In summary, venous thrombosis can progressively worsen DAVF with its concomitant symptoms such as coagulopathy and hypoxemia due to congenital heart failure.

**Table 1:** Dural arteriovenous fistula cases with Down syndrome.

Authors	Age, sex	Location of DAVF	Sinus thrombosis	History of craniotomy	Time of progress	Clinical presentation	Treatment	Cognard type
Koduri et al. [3]	14, F	IJV, SS	−	+	4 months	Asymptomatic	Observation	I
Berryman et al. [14]	46, F	SS	+	−	1 year	Headache, seizure	TAE	IV
Kim et al. [15]	20, M	TS-SS	+	−	Several months	Headache, nausea, blurred vision	TVE	IV
Our case	48, M	StS	+	+	2 years	Altered consciousness, hemiparesis	TAE	III

DAVF, dural arteriovenous fistula; IJV, internal jugular vein; SS, sigmoid sinus; StS, straight sinus; TAE, trans arterial embolization; TS, transverse sinus; TVE, transvenous embolization.

A progressive type of DAVF presenting with prominent neurological symptoms, such as intracranial hemorrhage, and convulsions is complicated with or preceded by venous thrombosis [4,9,13]. Our patient presented with intracranial hemorrhage, de novo DAVF, and venous thrombosis symptoms that appeared within 2 years of the craniotomy for brain abscesses. The development of these conditions in the brain was apparently induced by a local venous reflux disorder. Additionally, the patient's medical history included chronic hypoxemia and right heart failure, and these conditions may have promoted the angiogenesis. Further deterioration of venous congestion due to venous thrombosis has led to the progression of DAVF (Fig. 3) [2,4,6,9,11,12]. The result of TAE was successful for occlusion and shunt flow dismissal. However, the patient's consciousness was persistently altered because of irreversible damage to the bilateral thalamus by venous congestion from the early period of the clinical course, despite of the DAVF shunt flow occlusion. Thus, prompt recognition and early treatment of DAVF before the changes due to the venous reflux could prevent further irreversible damage.

To the best of our knowledge, this is the first case of de novo DAVF occurring within 2 years of craniotomy for brain abscesses in a patient with Down syndrome and Eisenmenger syndrome. DAVF could be a source of intracranial hemorrhage in Down syndrome cases. When concomitant symptoms such as hypoxemia due to congenital heart failure, and coagulopathy are present, the DAVF disease state can worsen progressively. Therefore, DAVF with Down syndrome needs to be monitored and treated carefully and thoroughly.

## Patient consent

Written informed consent was obtained from the patient's mother and sister to publish the details of their medical case and any accompanying images. Additionally, we obtained permission for this report from the ethical committee at our institute (YCU Center for Novel and Exploratory Clinical Trials, Y-NEXT B191200034).
